# Homogeneous sulfur isotope signature in East Antarctica and implication for sulfur source shifts through the last glacial-interglacial cycle

**DOI:** 10.1038/s41598-019-48801-1

**Published:** 2019-08-27

**Authors:** Sakiko Ishino, Shohei Hattori, Joel Savarino, Michel Legrand, Emmanuelle Albalat, Francis Albarede, Susanne Preunkert, Bruno Jourdain, Naohiro Yoshida

**Affiliations:** 1 Department of Chemical Science and Engineering, School of Materials and Chemical Technology, Tokyo Institute of Technology, 226-8502 Yokohama, Japan; 2 Institut des Geoscience de l’Environnement, Université Grenoble Alpes/CNRS, G-INP, IRD, 38000 Grenoble, France; 3 Ecole Normale Supérieure (LGL-TPE), 69364 Lyon, France; 4 Earth-Life Science Institute, Tokyo Institute of Technology, 152-8551 Tokyo, Japan; 5 Present Address: National Institute of Polar Research, 190-8518 Tokyo, Japan

**Keywords:** Element cycles, Atmospheric chemistry, Cryospheric science, Palaeoclimate

## Abstract

Sulfate aerosol (SO_4_^2−^) preserved in Antarctic ice cores is discussed in the light of interactions between marine biological activity and climate since it is mainly sourced from biogenic emissions from the surface ocean and scatters solar radiation during traveling in the atmosphere. However, there has been a paradox between the ice core record and the marine sediment record; the former shows constant non-sea-salt (nss-) SO_4_^2−^ flux throughout the glacial-interglacial changes, and the latter shows a decrease in biogenic productivity during glacial periods compared to interglacial periods. Here, by ensuring the homogeneity of sulfur isotopic compositions of atmospheric nss-SO_4_^2−^ (δ^34^S_nss_) over East Antarctica, we established the applicability of the signature as a robust tool for distinguishing marine biogenic and nonmarine biogenic SO_4_^2−^. Our findings, in conjunction with existing records of nss-SO_4_^2−^ flux and δ^34^S_nss_ in Antarctic ice cores, provide an estimate of the relative importance of marine biogenic SO_4_^2−^ during the last glacial period to be 48 ± 10% of nss-SO_4_^2−^, slightly lower than 59 ± 11% during the interglacial periods. Thus, our results tend to reconcile the ice core and sediment records, with both suggesting the decrease in marine productivity around Southern Ocean under the cold climate.

## Introduction

Secondary sulfate plays an important role in aerosol and cloud interactions and influences solar radiation^1^. In Antarctica, because of isolation from major anthropogenic SO_2_ emissions over the continents, the main source of non-sea-salt (nss-) SO_4_^2−^ is dimethyl sulfide (DMS) produced by marine phytoplankton living in the Southern Ocean^2^. Therefore, the nss-SO_4_^2−^ preserved in Antarctic ice cores is used as a record of past marine biogenic activity, and its response and feedback to climate change are debated^3–5^.

It has been shown that nss-SO_4_^2−^ flux recorded in Antarctic ice cores has not significantly changed throughout the last eight glacial cycles, which is concluded to indicate a nonsignificant change in marine biogenic activity^3^. However, this conclusion is inconsistent with the implication derived from marine sediment cores that shows lower productivity at latitudes higher than 50°S during the last glacial period than during the current warm period^6^. To unravel the cause of this paradox, identification of the sulfur sources of those nss-SO_4_^2−^ preserved in ice cores potentially provides helpful insights. The stable sulfur isotopic composition of nss-SO_4_^2−^ (δ^34^S_nss_) is a potential tool for quantitative estimates of the relative importance of marine biogenic (mb-) and nonmarine biogenic (nmb-) SO_4_^2−^. Indeed, the δ^34^S_nss_ values in snow and ice in East Antarctica^7–10^ have provided estimates of 80–90% dominance of mb-SO_4_^2−^ in nss-SO_4_^2−^ for the last several hundred years.

In addition to the dependence on its sulfur sources, the δ^34^S signature could be modified by isotopic fractionation via oxidation of SO_2_ to SO_4_^2−^. Although Alexander *et al*.^11^ observed the δ^34^S_nss_ values in deep ice cores that showed ca. 4‰ lower values during the last glacial period than during the current warm period, they interpreted those δ^34^S_nss_ values to be the result of significant isotopic fractionation through SO_2_ oxidation, which caused progressive washing out of isotopically heavier SO_4_^2− ^^12^ and transport of the remaining SO_2_ with low δ^34^S_nss_ values to inland. On the other hand, Uemura *et al*.^7^ examined remarkably uniform δ^34^S_nss_ (14–17‰) in surface snow over the transect between Syowa Station (69°00′S, 39°35′E) and Dome F (77°19′S, 39°42′E), concluding the absence of considerable isotopic fractionation during transport under present-day climate. However, surface snow observations are not sufficient to draw firm conclusions on the reasons for this homogeneity, as inhomogeneous snow deposition throughout the year and remobilization of snow by wind mask real atmospheric spatial and temporal variations. It is thus necessary to observe the differences in this signature between inland and coastal sites before the deposition of SO_4_^2−^, i.e., for SO_4_^2−^ in atmospheric aerosol samples, to clarify the mechanisms that cause constant flux throughout the ice ages.

To address the above discussion, we performed a year-round observation of δ^34^S_nss_ values of atmospheric SO_4_^2−^ at Dome C (75°06′S, 123°12′E; 3233 m a.s.l.) and Dumont d’Urville Station (DDU) (66°40′S, 140°01′E; 40 m a.s.l.), inland and coastal sites in East Antarctica, respectively, by utilizing continuous aerosol samples at those sites. Furthermore, we utilized δ^34^S_nss_ values to estimate changes in sulfur sources in both the present and the past Antarctic atmosphere to infer the major nonmarine sulfur sources that remain to be identified.

## Results

**Figure 1:**
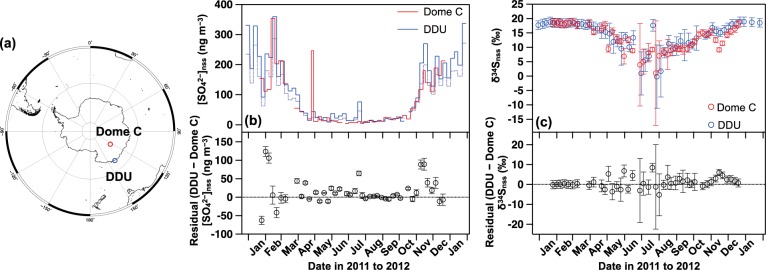
Observed concentrations and δ^34^S values of non-sea-salt (nss-) SO_4_^2−^. (**a**) Map of the aerosol sampling sites, with seasonal variations in (**b**) concentrations (solid line: total suspended particle, dashed line: fine mode particle) and (**c**) δ^34^S values of nss-SO_4_^2−^ at Dome C (red) and Dumont d’Urville (blue) with residual values between the two sites. Error bars represent the standard errors propagated from the analytical error of concentrations, δ^34^S values, and the uncertainty in SO_4_^2−^/Na^+^ ratios in sea salt (see *Methods*).

Figure 1 shows the time series of concentrations and δ^34^S values of SO_4_^2−^ at Dome C and DDU throughout 2011. Both were corrected into nss-SO_4_^2−^ values as described in the *Methods*. At both sites, nss-SO_4_^2−^ concentrations ([SO_4_^2−^]_nss_) show well-marked seasonality with maxima of up to ca. 300 ng m^−3^ in late austral summer (February), with minima less than 20 ng m^−3^ during winter (August) (Fig. 1b). These trends are consistent with continuous observations at Dome C^13,14^ and DDU^15,16^, which are known as a result of enhanced production of biogenic DMS over the Southern Ocean during the austral summer and its subsequent oxidation into SO_4_^2−^.

Along with the seasonal cycle in concentrations, the δ^34^S_nss_ values also show strong seasonality with the summer maxima and winter minima (Fig. 1b and Supplementary Table S2). During January–March, the δ^34^S_nss_ values stay within the narrow range of 17.7 to 18.6‰ at both sites (*n* = 21). In April, the δ^34^S_nss_ values decrease gradually and reach their lowest values during June–August with mean values of 8.2 ± 2.4‰ (*n* = 12) and 9.4 ± 2.6‰ (*n* = 11) at Dome C and DDU, respectively. The δ^34^S_nss_ values then increase during August–December, with a considerable decrease in November at Dome C, in contrast to the gradual change observed at DDU. This specific November trend at Dome C is discussed below. Consequently, the seasonal δ^34^S_nss_ cycles and their amplitudes are quite consistent between the two sites.

## Discussion

### Homogeneity in sulfur isotopic compositions of sulfate in the atmosphere

Figure 1b shows the difference in δ^34^S_nss_ values between the two sites by subtracting the δ^34^S_nss_ value of each Dome C sample from that of each DDU sample collected in the closest time period. It is obvious that most of the values do not statistically deviate from 0‰ within the propagated analytical error. Excluding the specifically low δ^34^S_nss_ values at Dome C in November, the residual values exhibit no systematic trend and averaged 0.5 ± 2.6‰. The δ^34^S_nss_ values were thus surprisingly homogeneous between Dome C and DDU throughout the year, ensuring that the isotopic fractionation for δ^34^S_nss_ during transport towards inland is far smaller than the observed variability ranging from 8.2 to 18.6‰. Moreover, the annual weighted averages of the δ^34^S_nss_ values were 15.8 ± 0.5‰ and 17.0 ± 0.4‰ for Dome C and DDU (Table S2), respectively, which are consistent with the δ^34^S_nss_ values of 13.7–16.6‰ observed for surface snow over the transect between Syowa Station and Dome Fuji by Uemura *et al*.^7^. This result indicates that the δ^34^S_nss_ signature is preserved in snow SO_4_^2−^ without any significant postdepositional effect.

This result suggests that the δ^34^S_nss_ values in the Antarctic atmosphere are not significantly influenced by isotopic fractionation during transport and are therefore controlled by the relative importance of sulfur sources. Fifteen years after advocating the possible change in the signature by isotopic fractionation^11^, these results bring back the applicability of the δ^34^S_nss_ signature for reconstruction of sulfur sources, i.e., the relative importance of mb-SO_4_^2−^ and nmb-SO_4_^2−^, through the past climate changes.

### Seasonal variation in marine biogenic SO_4_^2−^ and nonmarine SO_4_^2−^ derived from δ^34^S_nss_

**Figure 2:**
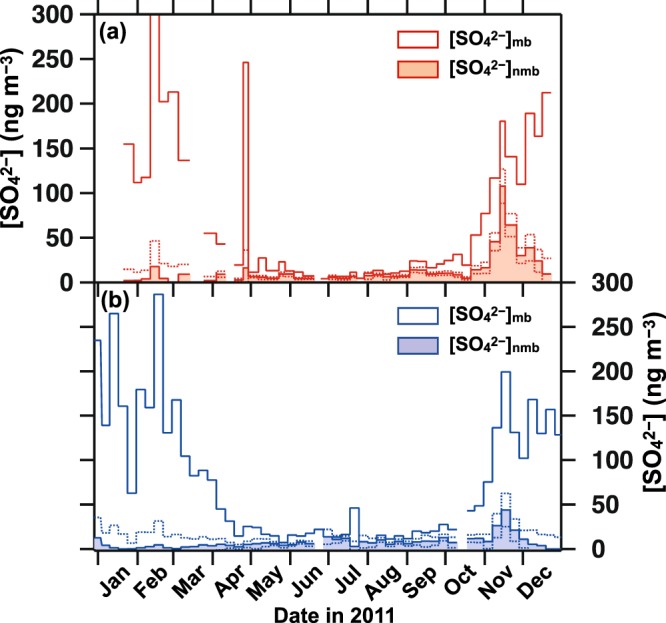
Estimated concentrations of mb-SO_4_^2−^ and nmb-SO_4_^2−^. (**a**) Dome C and (**b**) Dumont d’Urville with assuming δ^34^S_nmb_ = 2.5‰. Dotted lines represent the ranges of the uncertainty (standard error) for [SO_4_^2−^]_nmb_ propagated from the analytical error of concentrations and δ^34^S values.

Based on the above result, we utilized the signature to estimate the relative contribution of mb- and nmb-SO_4_^2−^ for the present atmospheric samples collected in this study (see *Methods* for the calculation process). Note that, for DDU samples, the estimate was applied to fine mode particle only due to the absence of δ^34^S_nss_ data for coarse mode particle (*Methods*), although the estimate was applied to total suspended particle for Dome C samples. As a result, the [SO_4_^2−^]_mb_ clearly shows strong seasonality with summer maxima and winter minima at both Dome C and DDU (Fig. 2), which correspond to 79–84% and 89–92% for annual total nss-SO_4_^2−^ at Dome C and DDU, respectively, and consequently controls the seasonality in [SO_4_^2−^]_nss_. By contrast, the [SO_4_^2−^]_nmb_ varies in a small range of 0–39 ng m^−3^ during most of the period throughout the year, except for November when the [SO_4_^2−^]_nmb_ increased significantly at both sites. Average values of [SO_4_^2−^]_nmb_ excluding November were 9.5 ± 7.7 ng m^−3^ and 5.9 ± 4.0 ng m^−3^ at Dome C and DDU, respectively.

**Figure 3:**
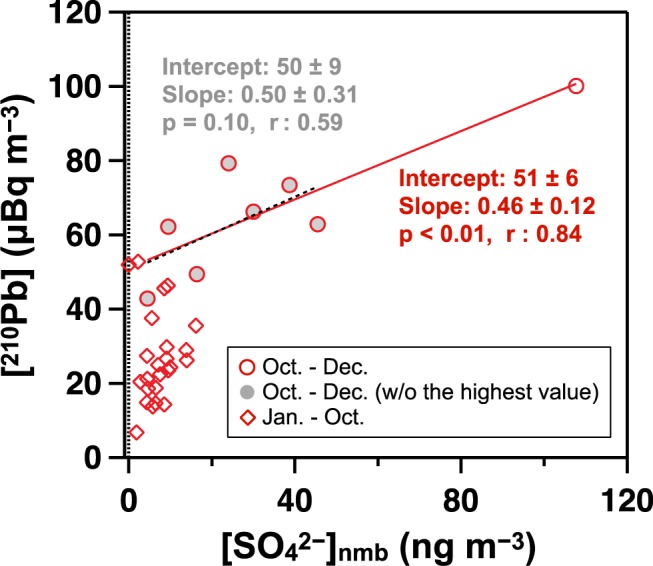
Relation between [^210^Pb] (μBq m^−3^) and [SO_4_^2−^]_nmb_ (ng m^−3^) at Dome C in 2011. Red circles represent data obtained during October–December, one month before and after the [SO_4_^2−^]_nmb_ increase in November. Grey circles and dotted line are the case without the highest [SO_4_^2−^]_nmb_. Data for other periods are presented as red diamonds.

In addition to such small variations during January–October, a marked increase in [SO_4_^2−^]_nmb_ up to 93–127 ng m^−3^ was observed in November at Dome C. Such an anomalous event was obscured by the increasing trends of mb-SO_4_^2−^ towards summer. The sum of nmb-SO_4_^2−^ during November accounts for ca. 50% of monthly nss-SO_4_^2−^ and ca. 10% of annual nss-SO_4_^2−^ at Dome C (Supplementary Fig. S1). Furthermore, the slight increase in [SO_4_^2−^]_nmb_ (38–52 ng m^−3^) was observed at DDU in exactly the same time period as that at Dome C, in the second week of November. Given the same timing at both sites and its higher amplitude at the inland site than at the coastal site, we first explored the possibility that this [SO_4_^2−^]_nmb_ increase is caused by nmb-SO_4_^2−^ emission located in inland Antarctica. However, this possibility is unlikely given that there has not been observed specific increases in volcanic activity of Mt. Erebus (77°53′S, 167°17′E), an active volcano on the Antarctic continent, during November 2011 based on satellite imagery data^17^. SO_2_ emissions from anthropogenic activities over Antarctica related to scientific activities are unlikely since they are generally highest during January–February^18^ and not in November. One attributable process is long-range transport of nmb-SO_4_^2−^ from other continents, which is supported by the significant correlation between our estimated [SO_4_^2−^]_nmb_ and previously observed ^210^Pb^14^, a commonly used tracer of continental submicron aerosols^19^, for the period from October to December with a *p*-value of less than 0.01 (Fig. 3 and Supplementary Table S3). Note that plausible sulfur sources of this specific [SO_4_^2−^]_nmb_ increase remained uncertain (detailed in Supplementary Information). Although this nmb-SO_4_^2−^ corresponds to only 10% of annual nss-SO_4_^2−^ in the present Antarctic atmosphere, future work to clarify its source is necessary for interpretation of the deep ice core record since it is suggested that the dominant sulfur source shifted to nmb-SO_4_^2−^ during the glacial period, as discussed in the next section.

### Implication for sulfur sources through glacial-interglacial changes

**Figure 4:**
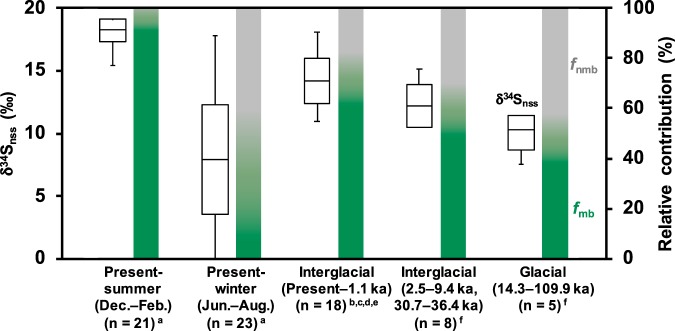
Observed δ^34^S_nss_ values in aerosol SO_4_^2−^ (^a^this study) and in snow and ice core SO_4_^2−^ (^b^Uemura *et al*.^7^, ^c^Jonsell *et al*.^8^, ^d^Baroni *et al*.^9^, ^e^Patris *et al*.^10^, ^f^Alexander *et al*.^11^) shown with the estimated relative contribution of marine biogenic (*f*_mb_, green) and nonmarine biogenic (*f*_nmb_, gray) SO_4_^2−^. Note that the δ^34^S_nss_ values in ice core samples with obvious volcanic inputs are excluded from this estimate. The box plot indicates the interquartile range (box) and the average (line), and the whiskers indicate minimum and maximum values.

We further applied the above calculation to estimate the shift in sulfur sources during glacial ages using the reported δ^34^S_nss_ values in snow and ice cores^7–11^ (Fig. 4). Alexander *et al*.^11^ reported δ^34^S_nss_ values in deep ice cores showing a decrease from 12.2 ± 1.8‰ in the warm periods (Holocene and Eemian) to 10.2 ± 1.6‰ in the last glacial period. These δ^34^S_nss_ values are clearly lower than our observation of 16.6 ± 0.3‰ on average for the present aerosol samples and 14.2 ± 1.8‰ for the other shallow ice cores. Such difference among the δ^34^S_nss_ values for interglacial samples is possibly because of volcanic influence as discussed in Supplementary Information, and here we consider δ^34^S_nss_ values reported by Alexander *et al*.^11^ as a representative for the interglacial values. This shift in δ^34^S_nss_ values corresponds to a gradual decrease in *f*_mb_ (the fraction of marine biogenic sulfate over the non-sea-salt sulfate) from 86 ± 3% at present to 59 ± 11% during the interglacial and to 48 ± 10% in the last glacial period (Fig. 4).

Thus, the δ^34^S_nss_ record in deep ice cores^11^ combined with the constant nss-SO_4_^2−^ flux record^3^ imply that marine biologically produced SO_4_^2−^ is decreased during the glacial period. This conclusion is more consistent with the marine sediment core records that show a decrease in biological carbon export production during glacial times around the vicinity of Antarctica at latitudes higher than 50°S^6^. As a consequence, our result tends to reconciliate conclusions drawn from ice core records and the marine sediment core records, with both suggesting a decrease in marine productivity around Antarctica during cold climates. At the same time, the estimated result shows that nmb-SO_4_^2−^ increased during the glacial period relative to the interglacial. Given that the mineral dust fluxes to Antarctica are controlled by changes in transport efficiency associated with the hydrological cycle^20^, it is likely that the increased nmb-SO_4_^2−^ during the glacial period was also sourced from other continents, although its sulfur source remains uncertain. The recent work based on the SO_4_^2−^ and Ca^2+^ ion concentrations over eight glacial cycles^21^ estimated that the proportional contributions of biogenic sulfur during cold periods could decrease to 24% or 52% in total nss-SO_4_^2−^, which depends on either assuming nss-Ca^2+^ originated only from primary terrestrial CaSO_4_ or from partial contribution of secondarily produced CaSO_4_ via the reaction between dust-sourced CaCO_3_ and marine biogenic sulfur. The latter case is in good agreement with our estimate of 48 ± 10% in the last glacial period (Fig. 4), suggesting the nss-SO_4_ during glacial periods may include both primary and secondary products.

Our estimate that the relative importance of nmb-SO_4_^2−^ had increased during the glacial period advances the understanding of the radiative cooling through the past climate change. Assuming that the micron-sized CaSO_4_ salt in Antarctic deep ice cores was secondarily produced during their transport from South America, Iizuka *et al*.^22^ concluded that the radiative cooling by marine biogenic sulfur had increased during the glacial periods, which is against the CLAW hypothesis proposed by Charlson *et al*.^23^. By contrast, our result suggests the sulfur source during glacial periods is not entirely marine biogenic but rather includes continental SO_4_^2−^. If CaSO_4_ during glacial period contained continental sulfur, those particles would have brought the radiative cooling to broad areas including a part of the continents in the mid-latitude of the Southern Hemisphere, while there would not have been influenced when assuming marine biogenic sulfur only. Therefore, the radiative cooling by sulfate may have been stronger than the previous estimate that had assumed a marine biogenic sulfur source alone^22^. Quantitatively, it is worth noting that *f*_nmb_ during inter-glacial and glacial periods were 41 ± 11% and 52 ± 10%, respectively, based on δ^34^S_nss_ (Fig. 4), and this difference is smaller than the relative increase in CaSO_4_ from 7 ± 4% during inter-glacial period to 56 ± 14% during glacial period^22^. Therefore, although it is true that the increased CaSO_4_ in the glacial period includes both primary and secondarily products, the changes in sulfur source should be taken into account since it would consequently affect the radiative forcing.

## Methods

### Sampling and ion quantification

Aerosol samples used for this study were collected in 2011 at Dome C (75°10′S, 123°30′E; 3233 m a.s.l.) and DDU (66°40′S, 140°01′E; 40 m a.s.l.). Sampling site details are presented in the Supplementary Information. Concentration data of Na^+^, MS^−^, Cl^−^, Br^−^, and C_2_O_4_^2−^ at Dome C were reported by Legrand *et al*.^14^. Additionally, all ion concentration data at DDU were reported by Ishino *et al*.^24^, where the sampling and quantification processes are detailed. Coarse (>1 μm) and fine (<1 μm) mode particles for DDU samples and total suspended particles for Dome C samples were collected using a high-volume air sampler at 1.5–1.7 m^3^ min^−1^ with a time resolution of 1–2 weeks at both sites. The aerosol loaded filters were kept frozen and were transported to Grenoble, where the ions were extracted to 40 mL of ultrapure water. Quantification of anions (NO_3_^−^, SO_4_^2−^) and cations (K^+^, Mg^2+^, Ca^2+^) was performed using the ion chromatography systems described by Savarino *et al*.^25^ and by Jourdain and Legrand^26^. The measured ion concentrations were corrected for blank values and were reported as the atmospheric concentration in standard temperature and pressure (*T* = 273.15 K, *p* = 101,325 Pa) based on meteorological data of Dome C and DDU provided by the IPEV/PNRA Project “Routine Meteorological Observation at Station Concordia” – (www.climantartide.it) and Meteo France. Uncertainties were estimated based on the typical uncertainty of the ion chromatography analyses (5%).

Concentrations of nss-SO_4_^2−^ were calculated by subtracting the sea salt fraction based on the Na^+^ concentration using the following equation, where *k* represents the [SO_4_^2−^]/[Na^+^] mass ratio in sea salt particles.

1 $${[{{\rm{SO}}}_{4}^{2-}]}_{{\rm{nss}}}={[{{\rm{SO}}}_{4}^{2-}]}_{{\rm{total}}}-k\times [{{\rm{Na}}}^{+}]$$

A *k* value in seawater of 0.25^27^ is generally used for this calculation. However, the sea salt emitted from the sea ice surface at low temperatures below −8 °C is depleted in SO_4_^2−^ relative to Na^+^ because of the precipitation of mirabilite^28^. The mixing of those sea salts emitted from the open ocean and sea ice surface results in *k* values at Dome C and DDU of 0.16 ± 0.09^29^ and 0.13 ± 0.04^26^, respectively. We applied those shifted values for samples collected during May–October.

### Sulfur isotope analyses

Sulfur isotopic compositions are expressed in delta notation defined with the following equation with respect to Vienna Canyon Diablo Troilite (VCDT) as a reference.

2 $${{\rm{\delta }}}^{34}{\rm{S}}=\frac{{({}^{{\rm{34}}}{\rm{S}}/{}^{{\rm{32}}}{\rm{S}})}_{{\rm{sample}}}}{{({}^{{\rm{34}}}{\rm{S}}/{}^{{\rm{32}}}{\rm{S}})}_{{\rm{reference}}}}-1$$

After ion quantification, samples were stored in a freezer at −20 °C before isotopic measurements. A total of 400 nmol and 2 μmol of SO_4_^2−^ were separated from other ions in each Dome C and DDU sample solution using ion chromatography, as described by Ishino *et al*.^24^. In this procedure, the yields of 100% of SO_4_^2−^ ensure no isotopic fractionation. We confirmed that the shifts in the δ^34^S values through this step were smaller than the analytical uncertainties in the subsequent procedures. Note that we analyzed the δ^34^S value for only the fine mode particles for the DDU samples because a large fraction of sea salt in the coarse mode particles (approximately 40% in summer to 100% in winter) leads to a large uncertainty. We used two methods for the sulfur isotope analyses of the Dome C and DDU samples because of sample size limitations.

For the Dome C samples, the δ^34^S values of sulfate were then measured using a multiple-collector inductively coupled plasma mass spectrometer (MC-ICP-MS; Neptune Plus, Thermo Fisher Scientific Inc.), as described by Albalat *et al*.^30^. Measurements were calibrated to values relative to VCDT with an external reproducibility of the in-house standard materials of ±0.12‰.

The DDU samples were processed through chemical conversion into Ag_2_S, as described in Geng *et al*.^31^, and then into SF_6_ in a method similar to that described by Ono *et al*.^32^, with modification as described by Hattori *et al*.^33^. We measured δ^34^S with a dual inlet system of an isotope ratio mass spectrometer (IRMS; Finnigan MAT 253, Thermo Fisher Scientific Inc.). The uncertainty in the measurement was estimated as ±0.2‰ based on replicate measurements of international standard materials (IAEA S1, S2, and NBS127).

The isotopic compositions of nss-SO_4_^2−^ for each sample were calculated with a simple mass balance equation with the sea salt SO_4_^2−^ fraction and δ^34^S_ss_ = 21.0‰^34^.

3 $${[{{\rm{SO}}}_{4}^{2-}]}_{{\rm{total}}}\,{{\rm{\delta }}}^{34}{{\rm{S}}}_{{\rm{total}}}={[{{\rm{SO}}}_{4}^{2-}]}_{{\rm{ss}}}\,{{\rm{\delta }}}^{34}{{\rm{S}}}_{{\rm{ss}}}+{[{{\rm{SO}}}_{4}^{2-}]}_{{\rm{nss}}}\,{{\rm{\delta }}}^{34}{{\rm{S}}}_{{\rm{nss}}}$$

Because of high sea salt loading on coarse mode particles at DDU, we used only fine mode particles for the isotope analyses.

### Sulfur source apportionment

The δ^34^S_nss_ values are determined by the relative contributions from various sulfur sources via the following equations:

4 $${{\rm{\delta }}}^{34}{{\rm{S}}}_{{\rm{nss}}}={\rm{\Sigma }}{f}_{i}\,{{\rm{\delta }}}^{34}{{\rm{S}}}_{i},$$

5 $${f}_{i}=\frac{{[{{\rm{S}}{\rm{O}}}_{4}^{2-}]}_{i}}{{[{{\rm{S}}{\rm{O}}}_{4}^{2-}]}_{{\rm{n}}{\rm{s}}{\rm{s}}}},$$

where *f*_*i*_, δ^34^S_*i*_ and [SO_4_^2−^]_*i*_ correspond to the relative contribution, the isotopic composition, and the concentration of sulfur source *i*, respectively. Here, we considered DMS emitted by marine biogenic activity (mb-), stratospheric SO_4_^2−^ inputs through vertical stratosphere-troposphere mixing or deposition of polar stratospheric clouds (st-), volcanic gaseous sulfur emissions (vl-), and anthropogenic sources, including those in the Antarctic continent and long-range transport from other continents (anth-), as possible sulfur sources^15^. Because the δ^34^S values of st-, vl-, and anth-SO_4_^2−^ are mutually overlapping, we designated them as nonmarine biogenic sources and assumed δ^34^S_nmb_ values as their sum (0 to 5‰)^35–37^. Since these δ^34^S_nmb_ values are distinguishable from the possible range of δ^34^S_mb_ values (16.6 to 20.3‰)^38–40^, the *f*_mb_ and *f*_nmb_ can be estimated by Eqs (4) and (5), with the assumption of mixing of the two endmembers. Note that it has been recently observed that biologically produced dimethylsulfoniopropionate (DMSP) in Antarctic sea ice possesses δ^34^S values largely ranging from 10.6 to 23.6‰, whose lowest values were observed in only the extreme physiochemical conditions of isolated brine pockets^40^. However, given that such low δ^34^S values are spatially limited and that the mean δ^34^S value of DMSP for the corresponding sea ice core sample was 17‰^40^, this sulfur source is unlikely to go beyond the range of general δ^34^S values of mb-SO_4_^2−^. The selection of δ^34^S_*i*_ values of each source and the validity of the assumption are discussed in detail in the Supplementary Information.

## Supplementary information

Supplementary Information

SI_Data_Ishino.xlsx
