# Supplementary material for: Homogeneous sulfur isotope signature in East Antarctica and implication for sulfur source shifts through the last glacial-interglacial cycle
Source: Sci Rep. 2019 Aug 27;9:12378. doi: 10.1038/s41598-019-48801-1 (PMC6711983; doi:10.1038/s41598-019-48801-1)
Supplement: Supplementary file 1 — Supplementary Information [file 41598_2019_48801_MOESM1_ESM.docx]

Supplementary Information for

**Homogeneous sulfur isotope signature in East Antarctica and implication for sulfur source shifts through the last glacial-interglacial cycle**

Sakiko Ishino^1,†,*^, Shohei Hattori^1,*^, Joel Savarino^2^, Michel Legrand^2^, Emmanuelle Albalat^3^, Francis Albarede^3^, Susanne Preunkert^2^, Bruno Jourdain^2^, and Naohiro Yoshida^1,4^

*Author Institution*: ^1^Department of Chemical Science and Engineering, School of Materials and Chemical Technology, Tokyo Institute of Technology, 226-8502 Yokohama, Japan; ^2^Institut des Geoscience de l’Environnement, Université Grenoble Alpes/CNRS, G-INP, IRD, 38000 Grenoble, France; ^3^Ecole Normale Supérieure (LGL-TPE), 69364 Lyon, France; ^4^Earth-Life Science Institute, Tokyo Institute of Technology, 152-8551 Tokyo, Japan

^†^now at National Institute of Polar Research, 190-8518 Tokyo, Japan

^*^*Corresponding authors*:

Sakiko Ishino, 190-8518 Tachikawa, Japan, +81425120608, [ishino.sakiko@nipr.ac.jp](mailto:ishino.sakiko@nipr.ac.jp)

Shohei Hattori, 226-8502 Yokohama, Japan, +81459245506, hattori.s.ab@m.titech.ac.jp

**This PDF file includes:**

Supplementary Information text

Figs. S1 to S8

Tables S1 to S4

Captions for databases S1

References for SI reference citations

**Other supplementary materials for this manuscript include the following:**

Datasets S1

Supplementary Information Text

Methods

**Sampling sites**

Dome C (75°06′S, 123°21′E; 3233 m above sea level) is located on the East Antarctic Plateau 1100 km distant from the nearest coast, facing the Indian Ocean sector. Dome C is the site for ice core drilling at which deep ice core covering the ca. 740,000 years of atmospheric records were extracted under the framework of European Project for Ice Coring in Antarctica^1,2^. Aerosol sampling was initiated in 2006 to elucidate the dynamics and chemistry governing the variation of gaseous and chemical components preserved in ice cores^3,4^. The climate at Dome C is extremely cold and dry, with temperatures ranging approximately −70 to −25 °C during the year. The Sun culminates at 38° on December 21: it is permanently below the horizon from May 6 to August 9. Typical wind speed is ca. 3 m s^−1^, favoring the 180–210° wind sector^5^.

Dumont d’Urville station (DDU, 66°40'S, 140°01'E; 40 m a.s.l.) is on a small island, 1 km off the coast of Antarctica. The station is one of the nearest to Dome C among all coastal Antarctic stations. The climate at DDU is described by Konig-Langlo et al.^6^. Compared with other parts of coastal Antarctica, DDU is temperate, with temperatures of −30 to 5 °C during the year. Most parts of the island are free of snow. The sea ice disappears completely during the austral summer. Even at solstices, the Sun reaches close to the horizon. The annual mean surface wind speed is 9.5 m s^−1^, with no clear seasonal variation. Strong katabatic winds favoring the 120–160° wind sector (i.e., from Antarctic interior) dominate at DDU, isolating the marine boundary layer from lower latitude air masses above it. Actually, DDU is at the edge of the polar vortex. Strong zonal winds (> 50 m s^−1^) prevail above 10 km from late autumn through the end of winter.

**Uncertainty for sulfur isotopic compositions**

Isotope measurements for Dome C samples were taken using a multiple-collector inductively coupled plasma mass spectrometer (MC-ICP-MS; Neptune Plus, Thermo Fisher Scientific Inc.)^7^ for δ^33^S, δ^34^S, and Δ^33^S values. From each sample, 400 nmol of SO_4_^2−^ was extracted and used for measurements. The isotopic compositions of in-house Alfa Aesar standard material ((NH_4_)_2_SO_4_ solution) were calibrated relative to Vienna Canyon Diablo Troilite (VCDT) with reproducibility of 0.16, 0.12, and 0.15 ‰, respectively, achieved for δ^33^S, δ^34^S, and Δ^33^S values through replicated measurements^7^. The measurements for samples relative to the Alfa Aesar standard were calibrated to the values relative to VCDT.

Isotope measurements for DDU samples were taken using a dual inlet system of an isotope ratio mass spectrometer (IRMS; Finnigan MAT 253, Thermo Fisher Scientific Inc.) using the SF_6_ method^8,9^, which enabled us to measure δ^33^S, δ^34^S, δ^36^S, Δ^33^S, and Δ^36^S values. To ensure precision, 2 μmol of SO_4_^2−^ was extracted from each sample. For all samples and international standard materials (IAEA S2 and NBS 127), the measured values relative to the SF_6_ working standard at Tokyo Tech. were calibrated to the values relative to VCDT with the measurement of Ag_2_S standard IAEA S1. Measurement of another Ag_2_S standard, IAEA S2, was conducted to confirm the uncertainty through fluorination processes^9^. NBS 127, BaSO_4_ form standards which was derived from ocean water, was processed in the same manner with the sulfate samples (including SO_4_^2−^ reducing process)^10^ to assess the overall uncertainty of measurements. Our measurements of NBS 127 closely matched those of the reported isotope values of seawater sulfate^11^ (Table S1). Based on the standard deviation (1σ) of these NBS 127 measurements, the overall uncertainties for δ^33^S, δ^34^S, δ^36^S, Δ^33^S, and Δ^36^S values were estimated respectively as 0.1, 0.2, 0.5, 0.01, and 0.3‰.

Results and discussion

**Weighted averages of δ^34^S_nss_ values**

The weighted averages of δ^34^S_nss_ values at both Dome C and DDU were calculated using Eq. (S1) as

${\delta^{34}S}_{nss,avg}=\frac{\sum_{i}^{n} \delta^{34}S_{nss,i} \times\left[ \mathrm{SO}_{4}^{2-} \right]_{nss,i}}{\sum_{i}^{n} \left[ \mathrm{SO}_{4}^{2-} \right]_{nss,i}}$ , (S1)


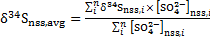


where *i* represents each sample and *n* represents the sample number of each time period of annual, seasonal, and monthly data. The results are presented in Table S2.

**Calculation for source apportionment**

Given that net isotopic fractionation during the transportation over Antarctica is within the difference between Dome C and DDU (0.5 ± 2.6‰), δ^34^S_nss_ values are determined by the relative contributions from various sulfur sources as shown in Eq. (S2):

$\left[ \mathrm{SO}_{4}^{2-} \right]_{\mathrm{nss}}\delta^{34}S_{\mathrm{nss}}=\sum{\left[ \mathrm{SO}_{4}^{2-} \right]_{i} \delta}^{34}S_{i}$ , (S2)

where [SO_4_^2−^]*_i_* and δ^34^S*_i_* respectively correspond to the concentration and the isotopic composition of sulfur source *i*. As described in the previous section, the major source of SO_4_^2−^ in the Antarctic atmosphere is DMS emitted from the surrounding ocean, which we designate here as mb-SO_4_^2−^, possessing specific δ^34^S values of 16.6 to 20.3‰^12-14^. Note that it has been recently observed that biologically produced dimethylsulfoniopropionate (DMSP) in Antarctic sea ice possesses the δ^34^S value largely ranging 10.6 to 23.6‰, whose lowest values were observed only in the extreme physiochemical conditions of isolated brine pocket^15^. However, given that such low δ^34^S values are limited and the mean δ^34^S value of DMSP for the corresponding sea ice core sample was 17‰^15^, this sulfur source is not likely to exceed the range of general δ^34^S values of mb-SO_4_^2−^.

Nmb-SO_4_^2−^ includes several different sources: terrigenous dust transported from other continents (tr-), stratospheric SO_4_^2−^ inputs through vertical stratosphere-troposphere mixing or deposition of polar stratospheric clouds (st-), volcanic gaseous sulfur emissions (vl-), and anthropogenic sources including those in the Antarctic continent and long-range transport from other continents (anth-)^16-20^. The δ^34^S_tr_ values are 0–20‰^21^ across the various regions, which is not distinct from mb-SO_4_^2−^. However, the relative contribution of tr-SO_4_^2−^ to nss-SO_4_^2−^ is expected to be less than 2% year-round, based on nss-Ca^2+^ concentration. It is therefore neglected in the following calculations. The δ^34^S_st_ values of background st-SO_4_^2−^ are reported to be identical over a wide range of latitudes in both the Northern and Southern Hemispheres, with the mean value of 2.6 ± 0.3‰^22^. The δ^34^S_vl_ value is commonly assumed to be 0–5‰ based on a series of observations of volcanic degassing^21^. Although no data of δ^34^S_anth_ around Antarctic regions are available, we used the δ^34^S_anth_ value by global shipping activity, which is usually assumed to be 3 ± 2‰^23,24^. Because these δ^34^S values of st-, vl-, and anth-SO_4_^2−^ are mutually overlapping, we assumed δ^34^S_nmb_ values as their sum (0 to 5‰) and used δ^34^S_nmb_ values of 0, 2.5, and 5‰ in the following calculations.

We denote the relative contributions of marine biogenic sulfate (*f*_mb_) and non-marine biogenic sulfate (*f*_nmb_) to non-sea salt sulfate for each sample as the following equations.

$f_{\mathrm{mb}}=\frac{\left[ \mathrm{SO}_{4}^{2-} \right]_{\mathrm{mb}}}{\left[ \mathrm{SO}_{4}^{2-} \right]_{\mathrm{nss}}}$ (S3)


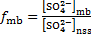


$f_{\mathrm{nmb}}=1-f_{\mathrm{mb}}$ (S4)


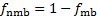


Results are presented in Fig. S1. At both Dome C and DDU, *f*_mb_ were almost completely 1 through December–March, showing the dominance of mb-SO_4_^2−^ persisting during the austral summer. However, *f*_nmb_ varied 30–100% during winter (May–September) at both sites. Therefore, nmb-SO_4_^2−^ accounts for a considerable fraction of nss-SO_4_^2−^ during winter, even though the actual amount of [SO_4_^2−^]_nmb_ varies only slightly and is almost constant throughout the year.

Considering mixing between mb-SO_4_^2−^ and nmb-SO_4_^2−^, Eq. (S5) can be converted into the following equation.

$\delta^{34}S_{\mathrm{nss}}= \delta^{34}S_{\mathrm{mb}}+ \frac{1}{{[\mathrm{SO}_{4}^{2-}]}_{\mathrm{nss}}}\times\left\{ \left[ \mathrm{SO}_{4}^{2-} \right]_{\mathrm{nmb}}\times\left( \delta^{34}S_{\mathrm{nmb}}-\delta^{34}S_{\mathrm{mb}} \right) \right\}$ (S5)

Eq. (S5) shows that δ^34^S_nss_ varies as a function of the inverse of [SO_4_^2−^]_nss_ under the assumption of constant values for δ^34^S_mb_, δ^34^S_nmb_, and [SO_4_^2−^]_nmb_. In fact, as the 1/[SO_4_^2−^]_nss_ approaches zero (i.e. the [SO_4_^2−^]_nss_ increases), the δ^34^S_nss_ values increase toward the intercepts of 16.9 ± 0.3‰ (Dome C) and 18.8 ± 0.3‰ (DDU) with marked linearity bearing *p*-values lower than 0.01 for each site (Fig. S2). The δ^34^S_nss_ values observed for Dome C from November 5 through December 3 are excluded from linear fitting. The significant linearity suggests that the assumption is reasonable: the nss-SO_4_^2−^ in the modern Antarctic atmosphere comprises two reservoirs, mb-SO_4_^2−^ and nmb-SO_4_^2−^, both bearing specific and distinct isotopic signatures, and [SO_4_^2−^]_nmb_ has small variation compared to [SO_4_^2−^]_mb_. The intercept values, which are within the range of δ^34^S_mb_ values, clearly reflect mb-SO_4_^2−^ dominance during summer time. The low intercept values for Dome C (16.9 ± 0.3‰) are probably attributable to the large fluctuation of winter δ^34^S_nss_ values caused by the variation in the contributions of different nmb-SO_4_^2−^ sources (Fig. 1c). Therefore, we applied the δ^34^S_mb_ values of 18.8 ± 0.3‰ derived from DDU data to Eq. (S2) to (S4) and estimated [SO_4_^2−^]_mb_ and [SO_4_^2−^]_nmb_ for both present atmospheric observation (Fig. 2 in the main text, Fig. S3) and deep ice core record (Fig. 4 in the main text).

**Possible source of significant nmb-SO_4_^2–^ increase in November 2011**

As mentioned in the main manuscript, the estimated [SO_4_^2−^]_mb_ for the present atmospheric samples show strong seasonal variation and consequently controls the seasonality in [SO_4_^2−^]_nss_ (Fig. 2). By contrast, the [SO_4_^2−^]_nmb_ showed characteristic variations at both sites with a small value during most of the period throughout the year and significant increase in November. Here we discuss the possible source of this specific [SO_4_^2−^]_nmb_ increase. Note that volcanic gaseous emission from Mt. Erebus (77°53′S, 167°17′E, 3794 m) is not likely the source of this specific increase given that no significant volcanic activity has been observed by satellite imagery data^25^. SO_2_ emissions from anthropogenic activities over Antarctica related to scientific activities are neither likely since they are generally highest during January–February^26^, not in November. Furthermore, the intrusion of stratospheric SO_4_^2–^ is neither likely considering the absence of specific increase in ^35^S in November^19^.

To explore the plausible sources of the [SO_4_^2−^]_nmb_ increase, we investigated the relation (correlation factors (*r*), *p*-values, and slopes) between [SO_4_^2−^]_nmb_ and other chemical species (^210^Pb, MS^−^, Cl^−^, Br^−^, NO_3_^−^, C_2_O_4_^2−^, Na^+^, NH_4_^+^, K^+^, Mg^2+^, Ca^2+^, O_3_) at each site during the time period of one month before and after the highest [SO_4_^2−^]_nmb_ (Table S3). For the calculation of non-sea salt fraction of Mg^2+^ ([Mg^2+^]_nss_ = [Mg^2+^] – m × [Na^+^]) and Ca^2+^ ([Ca^2+^]_nss_ = [Ca^2+^] – *n* × [Na^+^]), we used respectively the [Mg^2+^]/[Na^+^] ratio (m) and [Ca^2+^]/[Na^+^] ratio (*n*) in sea salt of 0.12 and 0.038^27^. Here we specifically discuss the correlation for the parameters with *p*-values lower than 0.05.

At Dome C, significant correlation with [SO_4_^2-^]_nmb_ was found for [^210^Pb] only (*r* = 0.84, *p* < 0.01). ^210^Pb, a radionuclide derived from the decay of crust-sourced ^222^Rn, has long been used to evaluate the long-range transport of continental sub-micron aerosols towards Antarctica^28^. Therefore, the significant correlation between [SO_4_^2-^]_nmb_ and [^210^Pb] (Fig. 3 in the main manuscript) allows us to consider the long-range transport of continental SO_4_^2-^ as a plausible source of November [SO_4_^2-^]_nmb_ increase.

At DDU, unfortunately the correlation between [SO_4_^2-^]_nmb_ and [^210^Pb] cannot be tested due to the lack of ^210^Pb observation. However, good positive correlations are found in [C_2_O_4_^2−^] (*r* = 0.90), [NH_4_^+^] (*r* = 0.78), [K^+^] (*r* = 0.78), those which are mainly sourced from ornithogenic soils at that time period ^29^. The best correlation with [Mg^2+^]_nss_ (*r* = 0.94) is probably also associated with ornithogenic emission. The ornithogenic SO_4_^2−^ contribution was estimated as reaching ca. 50 ng m^−3^ in November ^30^. Our estimate of maximum [SO_4_^2−^]_nmb_ at DDU during November is ca. 38–52 ng m^−3^ and might therefore be explained by the ornithogenic SO_4_^2−^ contribution, although it still remains uncertain because δ^34^S value of ornithogenic SO_4_^2−^ is unknown. Even if it were true, the [SO_4_^2−^]_nmb_ increase at Dome C requires other sources because the influence by penguins must be diluted at 1100 km distance from the coast despite the rather higher [SO_4_^2−^]_nmb_ (ca. 93–127 ng m^−3^) there. Therefore, we further step into the detailed discussion for possible sources of transported continental SO_4_^2–^.

Given that the [SO_4_^2−^]_nmb_ increase is an event for the period of only one month, it is reasonable to consider the possible influence of gaseous sulfur emission by volcanic eruption at the mid-latitude region of Southern Hemisphere. Indeed, there has been reported the eruption of Mt. Cerro Hudson (72.97°W, 45.90°S, 1905 m, Chile) in 26 October 2011 ^31^. However, it should be noted that the eruption products do not necessarily reach Dome C. While the eruption of Mt. Puyehue Cordon-Caulle (72.12°W, 40.59°S, 2236 m, Chile) in June 2011 resulted in considerable increase of SO_4_^2–^ in snow at West Antarctica^32^, there is no increase in [SO_4_^2−^]_nmb_ at Dome C during the same time period. Thus, the intrusion of eruption products of mid-latitude volcanoes requires both eruption event and atmospheric transport towards Antarctica.

Therefore, in order to test whether the sulfur sourced from the Mt. Cerro Hudson eruption in October could reach Dome C, we performed trajectory analysis using the Hybrid Single-Particle Lagrangian Integrated Trajectory model (available at: http://ready.arl.noaa.gov/HYSPLIT.php)^33^. We computed both 10-day backward trajectories departing from Dome C and 14-day forward trajectory departing from Mt. Cerro Hudson. Each trajectory starts at 0:00, 6:00, 12:00, and 18:00 UTC each day, with altitude of 20 m above ground level. Meteorological data from Global Data Assimilation Process (available at: ftp://arlftp.arlhq.noaa.gov/pub/archives/gdas1) were used as inputs. Fig. S4 shows trajectories arriving at Dome C during each of the aerosol sampling periods in November 2011. The air masses traveled around at latitudes higher than 70°S for most of the time (> 98%), and no noteworthy intrusion from the lower latitude regions was observed. By contrast, for the forward trajectory from Mt. Cerro Hudson, we found that the air mass starting at 0:00 of 29 October passed over the Antarctic sector between Dome C and DDU (Fig. S5, left), which might have transported volcanic sulfur to the vicinity of Dome C. It is noted however, that the highest [SO_4_^2−^]_nmb_ was observed in samples collected in the next period after this specific air mass (Fig. S5, right). As a result, we could not obtain clear evidence of the intrusion of eruption-sourced SO_4_^2–^ from the mid-latitude volcanoes.

We cannot rule out the possibility of the other sources for continental SO_4_^2–^. Considering that the increase of [^210^Pb] at Dome C happens in November for every year^34^, the increase in [SO_4_^2−^]_nmb_ might be a general trend rather than a specific event in that year. An earlier study showing long-term observations of [^210^Pb] at Neumayer Station (8.15°W, 70.39°S) ^28^ concluded that the seasonal cycle in [^210^Pb] is most likely controlled by a combination of the local change in the surface inversion persistence and seasonality in meridional long-range transport efficiency. Given similar seasonal trends in [^210^Pb] at Neumayer^28^ and at Dome C^34^, it is possible that the amplitude of November [SO_4_^2−^]_nmb_ at Dome C also depends on such atmospheric dynamics. If it were true, the source of the [SO_4_^2−^]_nmb_ increase might include regular emission from anthropogenic activity, non-eruptive outgassing from volcanoes, or sulfate contained aeolian dust, rather than short-term emission sources such as volcanic eruption. Inter-annual observation of δ^34^S_nss_ values of aerosol SO_4_^2−^ is necessary to further constrain the source of the specific increase of [SO_4_^2−^]_nmb_.

**Sulfur sources during winter**

Table S4 presents our estimates of [SO_4_^2−^]_nmb_ and *f*_nmb_ along with estimates obtained using different approaches. As explained above, earlier studies considered all or either of vl-, anth-, and st-SO_4_^2−^ as possible sources of nmb-SO_4_^2−^. The estimates of [SO_4_^2−^]_nmb_ and *f*_nmb_ are largely varied depending on the approaches and cannot be simply compared to our estimates. Nonetheless, two noticeable features exist: (i) especially in winter, estimates of *f*_nmb_ obtained using a chemistry-transport model (5.9–12.0% and 1.6–18.2% at Vostok (inland site) and DDU)^17^ are significantly lower than our estimates of *f*_nmb_ based on δ^34^S_nss_ (49–67% and 40–50% at Dome C and DDU); also, (ii) estimates of [SO_4_^2−^]_nmb_ using ^210^Pb tracer (20 and 8 ng m^−3^ at Dome C and 22 and 18 ng m^−3^ at DDU, respectively, for summer and winter) ^16,34^ are 2–10 times higher than our estimates of [SO_4_^2−^]_nmb_ based on δ^34^S_nss_ (< 11 ng m^−3^ at Dome C and < 9 ng m^−3^ at DDU throughout the year). Regarding feature (i), the difference between estimates of [SO_4_^2−^]_nmb_ obtained using the model and our δ^34^S_nss_ observation probably arises because of the lack of st-SO_4_^2−^ in the model, in addition to the too strong precipitation of SO_4_^2−^, which likely underestimates the contribution of long-range transport, as the authors described in their paper^17^. Estimates of [SO_4_^2−^]_st_ using ^35^S tracer at the inland site (5.5 and 3.0 ng m^−3^ in summer and winter, respectively)^19^ are also slightly lower than our estimates of [SO_4_^2−^]_nmb_ (> 5.9 and > 4.0 ng m^−3^ in summer and winter, respectively), suggesting that [SO_4_^2−^]_nmb_ is not solely explained by [SO_4_^2−^]_st_ and that it requires other sources. For feature (ii), Legrand et al.^34^ pointed out that the assumed SO_4_^2−^ concentration for the free troposphere of the South America region is probably overestimated because of the influence by cities close to the aerosol collection site that was chosen as representative of the South America region. Additionally, their study did not consider transportation from Australia, which is expected to be a major source of continental ^210^Pb to East Antarctica^35^. Given those uncertainties, estimates of [SO_4_^2−^]_nmb_ based on ^210^Pb are likely to be overestimates; also, [SO_4_^2−^]_nmb_ estimated based on δ^34^S_nss_ (this study) lower than them is apparently reasonable. The estimates of [SO_4_^2−^]_nmb_ based on MS^−^/SO_4_^2−^ ratio^34^ can be influenced not only by the relative contribution of mb-SO_4_^2−^ vs. nmb-SO_4_^2−^ but also by the DMS oxidation chemistry branching from production of MS^−^ and SO_4_^2−^. It is difficult to judge its reliability at this stage.

In summary, the consideration of st-SO_4_^2−^, vl-SO_4_^2−^, and anth-SO_4_^2−^ is likely to be reasonable for quantitative explanation of our estimated [SO_4_^2−^]_nmb_, although the influence by the long-range transport of nmb-SO_4_^2−^ from other continents represents considerable uncertainty.

**Evaluation of station contamination**

To evaluate the influence of station activity on sulfate in our samples, we defined the “contaminated time” (unit: %) as the time duration of hourly ozone concentration having been drastically diminished relative to the values obtained before and after because of the air-mass flow from the station to the observation site. For reference, the sampling duration of each sample was 5–10 days, i.e. 120–240 hr. The sum of the contaminated time for each sampling period was divided by the sampling duration and was compared to [SO_4_^2−^]_nss_ and δ^34^S_nss_ values (Fig. S6). We evaluated only winter (May–September) when [SO_4_^2−^]_nss_ is less than 30 ng m^−3^ at Dome C and DDU, i.e., when sulfate is presumed to be sensitive to contamination. We expected that if the station contamination had a marked impact on our sulfate samples, [SO_4_^2−^]_nss_ would increase and δ^34^S_nss_ values would approach δ^34^S_anth_ values (3 ± 2‰) as the contaminated time increases. As a result, no significant linearity was apparent in both [SO_4_^2−^]_nss_ and δ^34^S_nss_ values, indicating that the station contamination is not the important component of nss-SO_4_^2−^at both sites.

**Mass independent signatures: Δ^33^S and Δ^36^S values**

Mass-independent isotopic fractionation (MIF) signatures, Δ^33^S and Δ^36^S values, also provide insight into the formation processes of SO_4_^2−^. Although photoinduced reactions converting SO_2_ to SO_4_^2−^ in the stratosphere generates MIF^36^, background stratospheric SO_4_^2−^ produced from the reaction breaking down carbonyl sulfide (OCS) shows no evidence of sulfur MIF^37,38^. For that reason, large variations of MIF signatures found in SO_4_^2−^ in Antarctic ice or snow are thought to originate from Plinian volcanic eruptions that supplied huge amounts of SO_2_ to the stratosphere^39-42^. That SO_2_ photochemistry in the stratosphere leave a specific isotopic imprint in SO_4_^2−^ with the negative correlation between Δ^33^S and Δ^36^S values bearing the slope of −1.56 ± 0.25 with the intercept of 0.59 ± 0.21 (Fig. S7a)^42^.

Apart from the large MIF variation imprinted in stratospheric volcanic SO_4_^2−^, small variations of Δ^33^S and Δ^36^S values have been observed in tropospheric SO_4_^2−^ in the mid-latitude regions of the Northern Hemisphere (Fig. S7b)^43-46^. Recently, Lin et al.^46^ offered that the variations of MIF signatures in the tropospheric SO_4_^2−^ are likely to be explained by the partial contribution of stratospheric SO_4_^2−^ represented by the Archean reference lines (slope: −0.9 and −1.5)^36^ and primary SO_4_^2−^ produced via combustion processes^47^ in addition to the basis of mass dependent sulfur sources^46,48^, based on compiled Δ^33^S and Δ^36^S data in various regions.

In contrast, the Δ^33^S and Δ^36^S values of nss-SO_4_^2−^ in DDU aerosols in this study were, respectively, in narrow ranges of −0.08 to 0.07‰ and −1.2 to 0.9‰ (Fig. S7). Note that details of the MIF data for Dome C cannot be discussed because the Δ^33^S includes the analytical uncertainty of ±0.15‰ (Table S1) and because Δ^36^S was not measured ^7^ (*Methods*). Lack of correlation between Δ^33^S and Δ^36^S in DDU aerosols (*r* = −0.01) and its slope of −0.21, which is clearly different from the slope found for stratospheric volcanic SO_4_^2−^ (−1.56 ± 0.25) (Fig. S7a), reflects the absence of Plinian volcanic eruptions during 2011. Moreover, most Δ^33^S_nss_ values in DDU aerosols are within the range of possible variation in Δ^33^S induced by biogenic DMS production (up to 0.02‰) ^49^ from seawater SO_4_^2−^ (Δ^33^S = 0.048 ± 0.006‰)^11^ and SO_2_ oxidation occurring in the troposphere (± 0.10‰)^50^ (Fig. S7b). This result suggests that factors controlling MIF signatures observed in tropospheric SO_4_^2−^ in the Northern Hemisphere are processes of minor importance in Antarctica. Our dataset of SO_4_^2−^ in Antarctica with strictly mass-dependent isotopic signatures is expected to provide a better constraint on the mechanisms producing the MIF signatures of tropospheric SO_4_^2−^ in various regions worldwide in future works.

Fig. S7cd exhibit cross plots of δ^34^S_nss_ vs. Δ^33^S_nss_ values at both Dome C and DDU compared to values reported in earlier works. Data obtained from Antarctic ice cores for the stratospheric eruption events^39-42^ (Fig. S7c) have large variations with a positive relation between δ^34^S and Δ^33^S, bearing a specific slope of 0.09 ± 0.02^42^. By contrast, the Δ^33^S for Antarctic aerosols show insignificant variations (< ± 0.1‰) with no clear linearity between δ^34^S and Δ^33^S (*r* = 0.32 and 0.40 for Dome C and DDU, respectively), which is similar to non-volcanic sulfate in snow pits^41^ or fresh water^51^ in Antarctica. These results support our interpretation, based on Δ^33^S vs. Δ^36^S, that the SO_4_^2−^ in DDU samples was not influenced by stratospheric–volcanic SO_4_^2−^.

Fig. S7d shows that data for Antarctic aerosols are in a different range from those observed in the troposphere of other regions including Beijing^44^, Guangzhou^46^, and California^43^. Note that the small perturbation within ± 0.1‰ in Δ^33^S was not detectable for Dome C data due to analytical error of 0.15‰. The range of Δ^33^S values in those Northern Hemisphere regions of 0–0.5‰ is far larger than the range of Δ^33^S values at DDU (−0.08 to 0.07‰). The range of δ^34^S values in those regions of 0–10‰ is rather smaller and lower than those at DDU (0–19‰). Although the primary sulfate from combustion source (δ^34^S = 9–16‰, Δ^33^S = −0.2 to 0.0‰)^47^ can probably explain some of our Antarctic data, it cannot explain the δ^34^S values of 16–19‰ when the sulfur source is dominated by DMS during austral summer (Fig. 1 and Fig. 2 in the main text). This result emphasizes that the oxidation processes from DMS to SO_2_ and then to SO_4_^2−^ occurring around Antarctic regions are not correspondent to the MIF signatures observed in SO_4_^2-^ in the Northern Hemisphere. On the other hand, volcanic ash observed over North America (δ^34^S = −9 to 13‰, Δ^33^S = −0.07 to 0.01‰)^48^ shows no marked MIF signature. Consequently, volcanic sulfur also remains valid as a source of nmb-SO_4_^2−^, which engenders lower δ^34^S values during winter.

**Difference in δ^34^S_nss_ values in snow and ice core samples of interglacial periods**

There are slight differences in δ^34^S_nss_ values among the interglacial samples as shown in Fig. 4 in the main text. Compared to the mean δ^34^S_nss_ value of 16.6 ± 0.3 ‰ for our aerosol SO_4_^2−^ samples, the mean δ^34^S_nss_ values for snow and shallow ice core samples (14.2 ± 1.8 ‰)^20,23,40,52^ as well as for deep ice core samples of interglacial periods (12.2 ± 1.8 ‰)^53^ are slightly lower. These difference in δ^34^S_nss_ values for interglacial samples are likely the result of the different sample treatment for removal of volcanic SO_4_^2−^ at isotope analysis.

In the first report of δ^34^S values in the firn core at South Pole by Patris et al.^23^, the samples were cut with time resolution of 2–7 years and one sample covering the period of significant volcanic input by Mt. Agung eruption in 1963 was separated from other samples background SO_4_^2−^. This sample treatment enabled them to observe δ^34^S value of background SO_4_^2−^ ranging 14.8–18.1 ‰ and that with volcanic influence resulting lower δ^34^S value of 9.3 ‰. Similarly, Baroni et al.^40^ analyzed δ^34^S values for background and volcanic SO_4_^2−^ separately by identifying volcanic SO_4_^2−^ peaks based on SO_4_^2−^ concentration data. Also, Jonsell et al.^52^ analyzed the shallow ice core samples with excluding several samples with relatively high SO_4_^2−^ concentration. We applied those reported δ^34^S_nss_ values for background SO_4_^2−^ to the calculation of the mean δ^34^S_nss_ value for interglacial period and then obtained 14.2 ± 1.8 ‰ (Fig. 3 in the main text).

By contrast to the above reports, Alexander et al.^53^ did not apply such sample separation. They had concluded that the volcanic SO_4_^2−^ was probably not the major source of SO_4_^2−^ in their deep ice core samples, since the only five potential volcanic peaks were found for the period of 60–160 ka^54^ at that time and also the volcanic disturbances span only short time periods (2 years)^55^ relative to time resolution of their samples (50–100 years). However, the recent study analyzing SO_4_^2−^ concentration for both Greenland and Antarctic ice cores shows that there are 143 volcanic sulfate peaks for the sum of bipolar and Southern Hemisphere eruption through the past 2500 years ^56^. Assuming the same frequency of volcanic peaks for the older period, the ice core sample with 50–100 years resolution would contain at least 3 volcanic peaks for each. Thus, the volcanic influence for the deep ice core analyzed by Alexander et al.^53^ would have been larger than the authors originally thought.

Considering that the δ^34^S_nss_ values for the glacial period reported in Alexander et al.^53^ was obtained from the samples not separated from the volcanic SO_4_^2−^ influence, we regard the δ^34^S_nss_ values for the interglacial periods in the same work as appropriate for comparison between glacial-interglacial periods.

Fig. S1. Relative contributions of marine biogenic sulfate (*f*_mb_, strong color) and non-marine biogenic sulfate (*f*_nmb_, light color) at (a) Dome C and (b) DDU, when assuming δ^34^S_nmb_ = 2.5‰.

**Fig. S2.** Scatter plots of δ^34^S_nss_ values and the inverse of nss-SO_4_^2−^ concentrations for (a) Dome C and (b) Dumont d’Urville. Data obtained during November were excluded for Dome C.

Fig. S3. Concentrations of mb-SO_4_^2−^ and nmb-SO_4_^2−^ at (a,c) Dome C and (b,d) Dumont d’Urville, when assuming (a,b) δ^34^S_nmb_ = 0‰ and (c,d) δ^34^S_nmb_ = 5‰. Dotted lines represent the ranges of uncertainty for [SO_4_^2−^]_nmb_ propagated from the analytical error.

Fig. S4. 10-day backward trajectories starting from Dome C during each aerosol sampling period in November 2011. 13–17 November (up, right) corresponds to the time when [SO_4_^2−^]_nmb_ was highest at Dome C.

Fig. S5. 14-day forward trajectory starting at Mt. Cerro-Hudson during (left) and after (right) the eruption on 26 October 2011. Also, 13–17 November (right) corresponds to the time period when [SO_4_^2−^]_nmb_ was highest at Dome C.


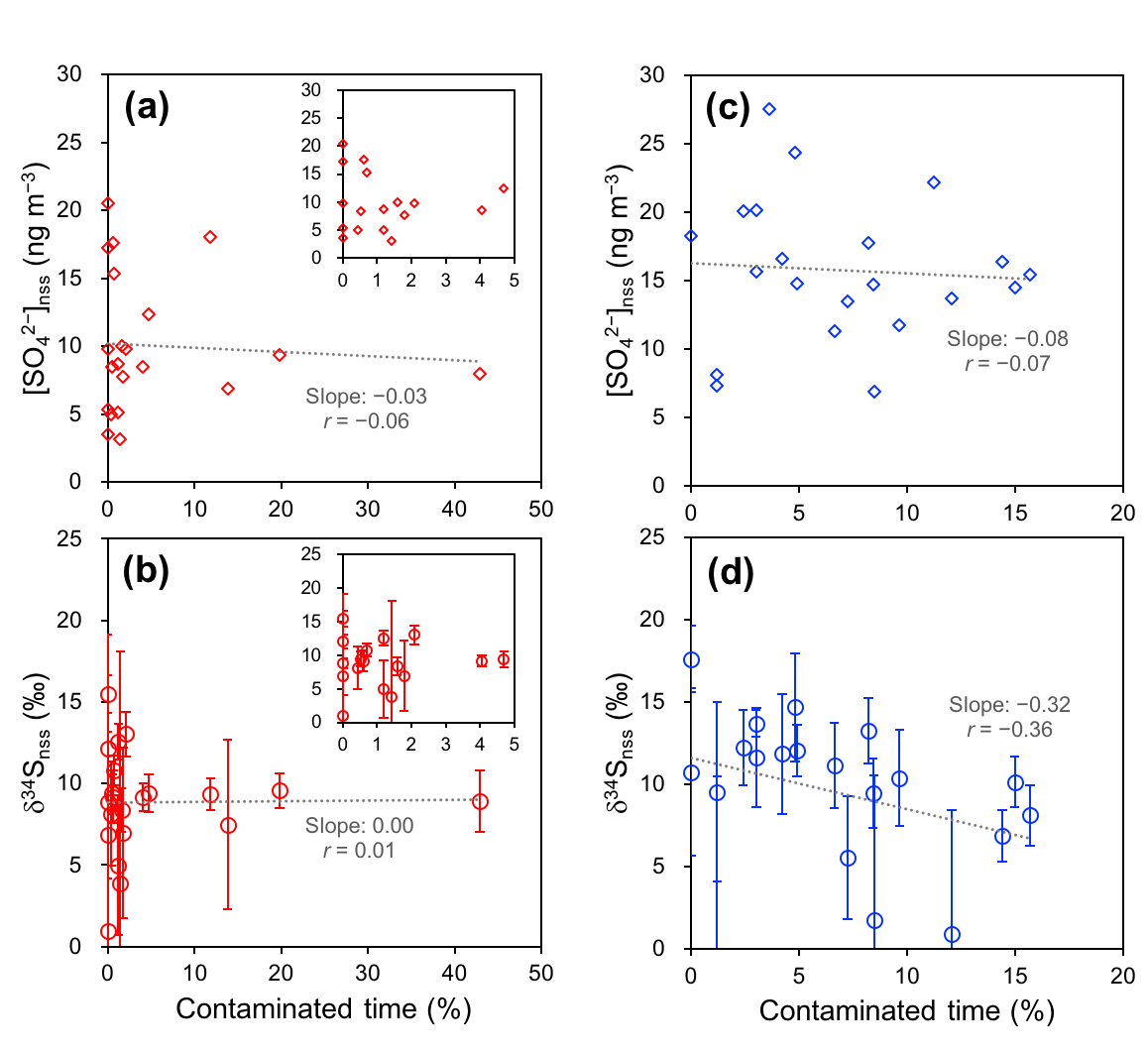


Fig. S6. Variations of (a,c) [SO_4_^2−^]_nss_ and (b,d) δ^34^S_nss_ values as a function of the time during which the sampling sites might be contaminated by station activity for (a,b) Dome C and (c,d) DDU. Only winter (May–September) data are examined.

Fig. S7. (Top) Cross plots of Δ^33^S_nss_ and Δ^36^S_nss_ values of DDU (blue open circles) compared to reported values for (a) Antarctic samples ^39-42,51^ and (b) present tropospheric SO_4_^2−^ samples ^11,36,43-50^. (bottom) δ^34^S_nss_ versus Δ^33^S_nss_ values of Dome C (red open circles) and DDU (blue open circles) compared to reported values for (c) Antarctic samples ^39-42,51^ and (d) the present tropospheric sulfate samples ^11,36,43-48^.

Table S1. Replicated standard measurements and correction of the values relative to V-CDT. For isotopic compositions in IAEA S1, we referred data from Ono et al. ^8^.


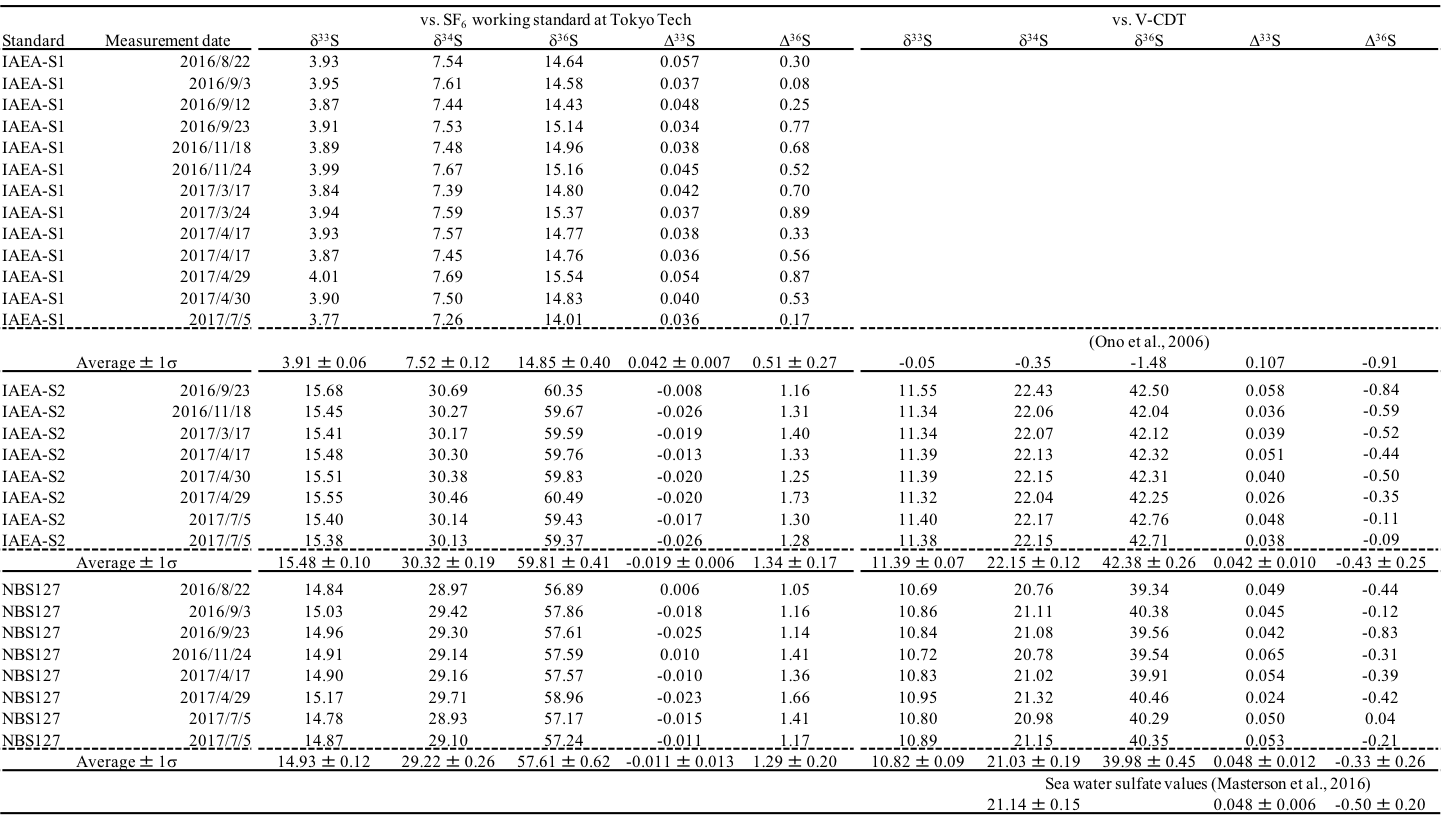


Table S2. Weighted average of δ^34^S_nss_ values for annual, seasonal, and monthly scale (units: ‰, except for sample number (*n*)). S.D. (1σ) were calculated using the median δ^34^S_nss_ values of each sample without considering the analytical error in each concentration and isotope data. “Error” for each averaged value was calculated by propagating the analytical error.

|  |  | Dome C | | | |  | Dumont d’Urville | | | |
| --- | --- | --- | --- | --- | --- | --- | --- | --- | --- | --- |
|  |  | value | S.D. (1σ) | Error | *n* |  | value | S.D. (1σ) | Error | *n* |
| Annual |  | 15.8 | 0.5 | 0.4 | 44 |  | 17.0 | 0.4 | 0.4 | 50 |
| Summer  (Dec–Feb) |  | 17.7 | 0.8 | 0.7 | 8 |  | 18.4 | 0.4 | 0.6 | 13 |
| Autumn  (Mar–May) |  | 17.3 | 0.8 | 0.8 | 11 |  | 17.3 | 0.5 | 0.8 | 13 |
| Winter (Jun–Aug) |  | 8.2 | 2.4 | 1.4 | 12 |  | 9.4 | 2.6 | 1.1 | 12 |
| Spring (Sep–Nov) |  | 12.0 | 1.3 | 0.4 | 13 |  | 14.9 | 0.8 | 0.8 | 12 |
| January |  | 18.6 |  | 1.3 | 1 |  | 18.5 | 0.9 | 1.2 | 4 |
| February |  | 18.2 | 1.4 | 1.0 | 4 |  | 18.4 | 0.8 | 1.1 | 4 |
| March |  | 18.3 | 1.8 | 1.2 | 3 |  | 18.2 | 0.7 | 1.1 | 4 |
| April |  | 17.3 | 1.2 | 1.4 | 3 |  | 16.9 | 0.5 | 1.2 | 5 |
| May |  | 12.2 | 2.3 | 0.8 | 5 |  | 12.7 | 2.6 | 2.4 | 4 |
| June |  | 9.8 | 4.8 | 3.0 | 3 |  | 7.3 | 4.3 | 1.5 | 4 |
| July |  | 6.7 | 5.6 | 3.3 | 4 |  | 11.9 | 4.8 | 2.0 | 4 |
| August |  | 8.3 | 3.8 | 1.6 | 5 |  | 8.3 | 5.3 | 1.9 | 4 |
| September |  | 9.7 | 3.6 | 0.8 | 4 |  | 11.3 | 2.9 | 1.8 | 5 |
| October |  | 14.0 | 1.2 | 0.8 | 4 |  | 14.2 | 2.0 | 1.5 | 3 |
| November |  | 11.9 | 2.2 | 0.5 | 5 |  | 15.6 | 0.8 | 1.0 | 4 |
| December |  | 16.7 | 1.0 | 1.0 | 3 |  | 18.3 | 0.7 | 1.0 | 5 |

Table S3. Correlation factors (*r*), *p*-values, and slopes relative to [SO_4_^2−^]_nmb_ for samples during 14 October 2011 – 26 December 2011. Values for which *p*-values are lower than 0.05 are in red. N.d. means the data not determined.

|  | |  | | Dome C | | | | |  | DDU | | |
| --- | --- | --- | --- | --- | --- | --- | --- | --- | --- | --- | --- | --- |
|  | |  | | *r* | | *p*-value | | Slope  ([X]/[SO_4_^2−^]_nmb_  in nmol m^−3^) |  | *r* | *p*-value | Slope  ([X]/[SO_4_^2−^]_nmb_  in nmol m^−3^) |
| [SO_4_^2−^]_total_ |  | | 0.43 | | 0.22 | | 0.85 | |  | 0.43 | 0.21 | 1.90 |
| [SO_4_^2−^]_nss_ |  | | 0.44 | | 0.21 | | 0.87 | |  | 0.37 | 0.29 | 1.47 |
| [^210^Pb] |  | | **0.84** | | **0.01** | | **-** | |  | n.d. | n.d. | - |
| [MS^−^] ^a^ |  | | −0.37 | | 0.29 | | −0.03 | |  | −0.48 | 0.16 | −0.44 |
| [Cl^−^] ^a^ |  | | −0.24 | | 0.50 | | −0.01 | |  | **0.79** | **0.01** | **5.70** |
| [Br^−^] ^a^ |  | | 0.23 | | 0.53 | | 0.00 | |  | −0.10 | 0.78 | 0.00 |
| [NO_3_^−^] |  | | 0.29 | | 0.42 | | 0.74 | |  | −0.22 | 0.53 | −0.24 |
| [C_2_O_4_^2−^] ^a^ |  | | −0.19 | | 0.60 | | 0.00 | |  | **0.90** | **0.00** | **0.26** |
| [Na^+^] ^a^ |  | | −0.58 | | 0.08 | | −0.50 | |  | **0.74** | **0.01** | **7.08** |
| [NH_4_^+^] |  | | n.d. | | n.d. | | - | |  | **0.78** | **0.01** | **11.22** |
| [K^+^] |  | | 0.29 | | 0.42 | | 0.05 | |  | **0.78** | **0.01** | **1.70** |
| [Mg^2+^] |  | | −0.12 | | 0.75 | | −0.01 | |  | **0.84** | **0.00** | **2.99** |
| [Mg^2+^]_nss_ |  | | 0.29 | | 0.45 | | 0.01 | |  | **0.94** | **0.00** | **2.07** |
| [Ca^2+^] |  | | 0.54 | | 0.11 | | 0.14 | |  | 0.49 | 0.15 | 0.16 |
| [Ca^2+^]_nss_ |  | | 0.59 | | 0.07 | | 0.10 | |  | n.d. | n.d. | - |
| [O_3_] ^b^ |  | | 0.50 | | 0.14 | | - | |  | 0.40 | 0.25 | - |
| Data obtained from ^a^ Legrand et al. ^34^ and ^b^ Legrand et al. ^57^ | | | | | | | | | | | | |

**Table S4.** Contributions of non-marine biogenic sulfate (unit: ng m^−3^) estimated using various approaches. Values in parentheses are the relative contributions (*f*_nmb_, unit: %). Estimates from this study are shown as ranges arising from the assumption of δ^34^S_nmb_ values of 0–5‰.

Additional data Table S1 (separate file)

Ion concentrations (ng m^−3^) and sulfur isotopic compositions of SO_4_^2−^ (‰) in each aerosol sample and concentrations of ^210^Pb (μBq m^−3^) and ozone (ppbv). Estimated concentrations and relative contributions of mb-SO_4_^2−^ and nmb-SO_4_^2−^ are also shown.

**References**

1 Augustin, L. *et al.* Eight glacial cycles from an Antarctic ice core. *Nature* **429**, 623-628, doi:10.1038/nature02599 (2004).

2 Wolff, E. W. *et al.* Southern Ocean sea-ice extent, productivity and iron flux over the past eight glacial cycles. *Nature* **440**, 491-496, doi:10.1038/nature04614 (2006).

3 Preunkert, S. *et al.* Seasonality of sulfur species (dimethyl sulfide, sulfate, and methanesulfonate) in Antarctica: Inland versus coastal regions. *Journal of Geophysical Research* **113**, doi:10.1029/2008jd009937 (2008).

4 Jourdain, B. *et al.* Year-round record of size-segregated aerosol composition in central Antarctica (Concordia station): Implications for the degree of fractionation of sea-salt particles. *Journal of Geophysical Research* **113**, doi:10.1029/2007jd009584 (2008).

5 Argentini, S. *et al.* The surface layer observed by a high-resolution sodar at DOME C, Antarctica. *Ann Geophys-Italy* **56**, doi:10.4401/ag-6347 (2013).

6 König-Langlo, G., King, J. C. & Pettré, P. Climatology of the three coastal Antarctic stations Dumont d'Urville, Neumayer, and Halley. *Journal of Geophysical Research: Atmospheres* **103**, 10935-10946, doi:10.1029/97jd00527 (1998).

7 Albalat, E. *et al.* Sulfur isotope analysis by MC-ICP-MS and application to small medical samples. *Journal of Analytical Atomic Spectrometry* **31**, 1002-1011, doi:10.1039/c5ja00489f (2016).

8 Ono, S., Wing, B., Johnston, D., Farquhar, J. & Rumble, D. Mass-dependent fractionation of quadruple stable sulfur isotope system as a new tracer of sulfur biogeochemical cycles. *Geochimica et Cosmochimica Acta* **70**, 2238-2252, doi:10.1016/j.gca.2006.01.022 (2006).

9 Hattori, S. *et al.* Determination of the sulfur isotope ratio in carbonyl sulfide using gas chromatography/isotope ratio mass spectrometry on fragment ions ^32^S^+^, ^33^S^+^, and ^34^S^+^. *Anal Chem* **87**, 477-484, doi:10.1021/ac502704d (2015).

10 Geng, L. *et al.* A simple and reliable method reducing sulfate to sulfide for multiple sulfur isotope analysis. *Rapid Commun Mass Spectrom* **32**, 333-341, doi:10.1002/rcm.8048 (2018).

11 Masterson, A. L., Wing, B. A., Paytan, A., Farquhar, J. & Johnston, D. T. The minor sulfur isotope composition of Cretaceous and Cenozoic seawater sulfate. *Paleoceanography* **31**, 779-788, doi:10.1002/2016pa002945 (2016).

12 Amrani, A., Said-Ahmad, W., Shaked, Y. & Kiene, R. P. Sulfur isotope homogeneity of oceanic DMSP and DMS. *Proc Natl Acad Sci U S A* **110**, 18413-18418, doi:10.1073/pnas.1312956110 (2013).

13 Oduro, H., Van Alstyne, K. L. & Farquhar, J. Sulfur isotope variability of oceanic DMSP generation and its contributions to marine biogenic sulfur emissions. *Proc Natl Acad Sci U S A* **109**, 9012-9016, doi:10.1073/pnas.1117691109 (2012).

14 Sanusi, A. A., Norman, A. L., Burridge, C., Wadleigh, M. & Tang, W. W. Determination of the S isotope composition of methanesulfonic acid. *Analytical Chemistry* **78**, 4964-4968, doi:10.1021/ac0600048 (2006).

15 Carnat, G. *et al.* Variability in sulfur isotope composition suggests homogenous dimethylsulfoniopropionate cycling and microalgae metabolism in Antarctic sea ice. *Communications Biology* **1**, 1-9, doi:10.1038/s42003-018-0228-y (2019).

16 Minikin, A. *et al.* Sulfur-containing species (sulfate and methanesulfonate) in coastal Antarctic aerosol and precipitation. *Journal of Geophysical Research: Atmospheres* **103**, 10975-10990, doi:10.1029/98jd00249 (1998).

17 Cosme, E., Hourdin, F., Genthon, C. & Martinerie, P. Origin of dimethylsulfide, non-sea-salt sulfate, and methanesulfonic acid in eastern Antarctica. *J Geophys Res-Atmos* **110**, D03302, doi:10.1029/2004jd004881 (2005).

18 Graf, H. F. *et al.* Continental scale Antarctic deposition of sulphur and black carbon from anthropogenic and volcanic sources. *Atmospheric Chemistry and Physics* **10**, 2457-2465, doi:10.5194/acp-10-2457-2010 (2010).

19 Hill-Falkenthal, J., Priyadarshi, A., Savarino, J. & Thiemens, M. Seasonal variations in35S and Δ17O of sulfate aerosols on the Antarctic plateau. *Journal of Geophysical Research: Atmospheres* **118**, 9444-9455, doi:10.1002/jgrd.50716 (2013).

20 Uemura, R. *et al.* Sulfur isotopic composition of surface snow along a latitudinal transect in East Antarctica. *Geophysical Research Letters* **43**, 5878-5885, doi:10.1002/2016gl069482 (2016).

21 Nielsen, H. *et al.* Lithospheric Sources of Sulphur. *Stable Isotopes in the Assessment of Natural and Anthropogenic Sulphur in the Environment*, 65-132 (1991).

22 Castleman, A. W., Munkelwitz, H. R. & Manowitz, B. Isotopic Studies of Sulfur Component of Stratospheric Aerosol Layer. *Tellus* **26**, 222-234, doi:10.1111/j.2153-3490.1974.tb01970.x (1974).

23 Patris, N., Mihalopoulos, N., Baboukas, E. D. & Jouzel, J. Isotopic composition of sulfur in size-resolved marine aerosols above the Atlantic Ocean. *Journal of Geophysical Research: Atmospheres* **105**, 14449-14457, doi:10.1029/1999jd901101 (2000).

24 Rempillo, O. *et al.* Dimethyl sulfide air-sea fluxes and biogenic sulfur as a source of new aerosols in the Arctic fall. *Journal of Geophysical Research* **116**, doi:10.1029/2011jd016336 (2011).

25 Global Volcanism Program, Report on Erebus (Antarctica). In: Venzke, E (ed.), *Bulletin of the Global Volcanism Network*, **42:6**. Smithsonian Institution (2017).

26 Shirsat, S. V. & Graf, H. F. An emission inventory of sulfur from anthropogenic sources in Antarctica. *Atmospheric Chemistry and Physics* **9**, 3397-3408, doi:10.5194/acp-9-3397-2009 (2009).

27 Holland, H. D., Lazar, B. & Mccaffrey, M. Evolution of the Atmosphere and Oceans. *Nature* **320**, 27-33, doi:10.1038/320027a0 (1986).

28 Elsässer, C. *et al.* Continuous 25-yr aerosol records at coastal Antarctica Part 2: variability of the radionuclides Be-7, Be-10 and Pb-210. *Tellus B* **63**, 920-934, doi:10.1111/j.1600-0889.2011.00543.x (2011).

29 Legrand, M., Ducroz, F., Wagenbach, D., Mulvaney, R. & Hall, J. Ammonium in coastal Antarctic aerosol and snow: Role of polar ocean and penguin emissions. *Journal of Geophysical Research: Atmospheres* **103**, 11043-11056, doi:10.1029/97jd01976 (1998).

30 Jourdain, B. & Legrand, M. Year-round records of bulk and size-segregated aerosol composition and HCl and HNO_3_ levels in the Dumont d'Urville (coastal Antarctica) atmosphere: Implications for sea-salt aerosol fractionation in the winter and summer. *J Geophys Res-Atmos* **107**, 4645, doi:10.1029/2002jd002471 (2002).

31 Global Volcanism Program, Report on Cerro Hudson (Chile). In: Wunderman, R (ed.), *Bulletin of the Global Volcanism Network*, 38:12. Smithsonian Institution. https://doi.org/10.5479/si.GVP.BGVN201312-358057 (2013).

32 Koffman, B. G. *et al.* Rapid transport of ash and sulfate from the 2011 Puyehue-Cordon Caulle (Chile) eruption to West Antarctica. *J Geophys Res-Atmos* **122**, 8908-8920, doi:10.1002/2017jd026893 (2017).

33 Stein, A. F. *et al.* Noaa's Hysplit Atmospheric Transport and Dispersion Modeling System. *B Am Meteorol Soc* **96**, 2059-2077, doi:10.1175/Bams-D-14-00110.1 (2015).

34 Legrand, M. *et al.* Year-round record of bulk and size-segregated aerosol composition in central Antarctica (Concordia site) – Part 2: Biogenic sulfur (sulfate and methanesulfonate) aerosol. *Atmospheric Chemistry and Physics* **17**, 14055-14073, doi:10.5194/acp-17-14055-2017 (2017).

35 Heimann, M., Monfray, P., & Polian, G. Modeling the long-range transport of 222Rn to subantarctic and Antarctic areas, *Tellus B* 42:83-89 (1990).

36 Ono, S. Photochemistry of Sulfur Dioxide and the Origin of Mass-Independent Isotope Fractionation in Earth's Atmosphere. *Annu Rev Earth Pl Sc* **45**, 301-329, doi:10.1146/annurev-earth-060115-012324 (2017).

37 Hattori, S. *et al.* Ultraviolet absorption cross sections of carbonyl sulfide isotopologues OC^32^S, OC^33^S, OC^34^S and O^13^CS: isotopic fractionation in photolysis and atmospheric implications. *Atmospheric Chemistry and Physics* **11**, 10293-10303, doi:10.5194/acp-11-10293-2011 (2011).

38 Schmidt, J. A. *et al.* OCS photolytic isotope effects from first principles: sulfur and carbon isotopes, temperature dependence and implications for the stratosphere. *Atmospheric Chemistry and Physics* **13**, 1511-1520, doi:10.5194/acp-13-1511-2013 (2013).

39 Savarino, J., Romero, A., Cole-Dai, J., Bekki, S. & Thiemens, M. H. UV induced mass-independent sulfur isotope fractionation in stratospheric volcanic sulfate. *Geophysical Research Letters* **30**, doi:10.1029/2003gl018134 (2003).

40 Baroni, M., Thiemens, M. H., Delmas, R. J. & Savarino, J. Mass-Independent Sulfur Isotopic Compositions in Stratospheric Volcanic Eruptions. *Science* **315**, 84-87, doi:10.1126/science.1131754 (2007).

41 Shaheen, R. *et al.* Large sulfur-isotope anomaly in nonvolcanic sulfate aerosol and its implications for the Archean atmosphere. *Proceedings of the National Academy of Sciences* **111**, 11979-11983, doi:10.1073/pnas.1406315111 (2014).

42 Gautier, E., Savarino, J., Erbland, J., & Farquhar, J. SO_2_ oxidation kinetics leave a consistent isotopic imprint on volcanic ice core sulfate. *J Geosphys Res: Atmos*, 123: 9801–9812. https:// doi.org/10.1029/2018JD028456 (2018).

43 Romero, A. B. & Thiemens, M. H. Mass-independent sulfur isotopic compositions in present-day sulfate aerosols. *J Geophys Res-Atmos* **108**, doi:10.1029/2003jd003660 (2003).

44 Guo, Z. *et al.* Identification of sources and formation processes of atmospheric sulfate by sulfur isotope and scanning electron microscope measurements. *Journal of Geophysical Research* **115**, doi:10.1029/2009jd012893 (2010).

45 Lin, M. *et al.* Atmospheric sulfur isotopic anomalies recorded at Mt. Everest across the Anthropocene. *Proceedings of the National Academy of Sciences* **115**, 6964-6969, doi:10.1073/pnas.1801935115 (2018).

46 Lin, M. *et al.* Five-S-isotope evidence of two distinct mass-independent sulfur isotope effects and implications for the modern and Archean atmospheres. *P Natl Acad Sci USA* **115**, 8541-8546, doi:10.1073/pnas.1803420115 (2018).

47 Lee, C. C. W., Savarino, J., Cachier, H. & Thiemens, M. H. Sulfur (S-32, S-33, S-34, S-36) and oxygen (O-16, O-17, O-18) isotopic ratios of primary sulfate produced from combustion processes. *Tellus B* **54**, 193-200, doi:10.1034/j.1600-0889.2002.01384.x (2002).

48 Bindeman, I. N., Eiler, J. M., Wing, B. A. & Farquhar, J. Rare sulfur and triple oxygen isotope geochemistry of volcanogenic sulfate aerosols. *Geochimica et Cosmochimica Acta* **71**, 2326-2343, doi:10.1016/j.gca.2007.01.026 (2007).

49 Oduro, H., Kamyshny, A., Guo, W. & Farquhar, J. Multiple sulfur isotope analysis of volatile organic sulfur compounds and their sulfonium precursors in coastal marine environments. *Marine Chemistry* **124**, 78-89, doi:10.1016/j.marchem.2010.12.004 (2011).

50 Harris, E., Sinha, B., Hoppe, P. & Ono, S. High-precision measurements of ^33^S and ^34^S fractionation during SO_2_ oxidation reveal causes of seasonality in SO_2_ and sulfate isotopic composition. *Environ Sci Technol* **47**, 12174-12183, doi:10.1021/es402824c (2013).

51 Kim, Y., Lee, I., Seo, J. H., Lee, J. I. & Farquhar, J. Multiple oxygen (^16^O, ^17^O and ^18^O) and sulfur (^32^S, ^33^S, ^34^S and ^36^S) isotope signatures of the dissolved sulfate from Deception Island, Antarctic Peninsula: Implications on sulfate formation, transportation and deposition in the Antarctic region. *Chemical Geology* **466**, 762-775, doi:10.1016/j.chemgeo.2017.07.029 (2017).

52 Jonsell, U., Hansson, M. E., Morth, C. M. & Torssander, P. Sulfur isotopic signals in two shallow ice cores from Dronning Maud Land, Antarctica. *Tellus B* **57**, 341-350, doi:10.1111/j.1600-0889.2005.00157.x (2005).

53 Alexander, B. *et al.* East Antarctic ice core sulfur isotope measurements over a complete glacial-interglacial cycle. *Journal of Geophysical Research: Atmospheres* **108**, 4876, doi:10.1029/2003jd003513 (2003).

54 Siegert, M. J., Hodgkins, R. & Dowdeswell, J. A. A chronology for the Dome C deep ice-core site through radio-echo layer correlation with the Vostok ice core, Antarctica. *Geophysical Research Letters* **25**, 1019-1022 (1998).

55 Legrand, M. R., Lorius, C., Barkov, N. I. & Petrov, V. N. Vostok (Antarctica) ice core: Atmospheric chemistry changes over the last climatic cycle (160,000 years). *Atmospheric Environment* **22**, 317-331 (1988).

56 Sigl, M. *et al.* Timing and climate forcing of volcanic eruptions for the past 2,500 years. *Nature* **523**, 543-549, doi:10.1038/nature14565 (2015).

57 Legrand, M. *et al.* Inter-annual variability of surface ozone at coastal (Dumont d'Urville, 2004–2014) and inland (Concordia, 2007–2014) sites in East Antarctica. *Atmospheric Chemistry and Physics* **16**, 8053-8069, doi:10.5194/acp-16-8053-2016 (2016).
